# Robustness of Eco-Epidemiological Capture-Recapture Parameter Estimates to Variation in Infection State Uncertainty

**DOI:** 10.3389/fvets.2018.00197

**Published:** 2018-08-28

**Authors:** Sarah Benhaiem, Lucile Marescot, Heribert Hofer, Marion L. East, Jean-Dominique Lebreton, Stephanie Kramer-Schadt, Olivier Gimenez

**Affiliations:** ^1^Department of Ecological Dynamics, Leibniz Institute for Zoo and Wildlife Research, Berlin, Germany; ^2^CEFE, CNRS, University Montpellier, University Paul Valéry Montpellier 3, EPHE, IRD, Montpellier, France; ^3^Department of Veterinary Medicine, Freie Universität Berlin, Berlin, Germany; ^4^Department of Biology, Chemistry, and Pharmacy, Berlin, Germany; ^5^Department of Ecology, Technische Universität Berlin, Berlin, Germany

**Keywords:** multi-event capture-mark-recapture, state uncertainty, partial observation, assignment probability, bias, precision, simulation, SIR model

## Abstract

Estimating eco-epidemiological parameters in free-ranging populations can be challenging. As known individuals may be undetected during a field session, or their health status uncertain, the collected data are typically “imperfect”. Multi-event capture-mark-recapture (MECMR) models constitute a substantial methodological advance by accounting for such imperfect data. In these models, animals can be “undetected” or “detected” at each time step. Detected animals can be assigned an infection state, such as “susceptible” (S), “infected” (I), or “recovered” (R), or an “unknown” (U) state, when for instance no biological sample could be collected. There may be heterogeneity in the assignment of infection states, depending on the manifestation of the disease in the host or the diagnostic method. For example, if obtaining the samples needed to prove viral infection in a detected animal is difficult, this can result in a low chance of assigning the I state. Currently, it is unknown how much uncertainty MECMR models can tolerate to provide reliable estimates of eco-epidemiological parameters and whether these parameters are sensitive to heterogeneity in the assignment of infection states. We used simulations to assess how estimates of the survival probability of individuals in different infection states and the probabilities of infection and recovery responded to (1) increasing infection state uncertainty (i.e., the proportion of U) from 20 to 90%, and (2) heterogeneity in the probability of assigning infection states. We simulated data, mimicking a highly virulent disease, and used SIR-MECMR models to quantify bias and precision. For most parameter estimates, bias increased and precision decreased gradually with state uncertainty. The probabilities of survival of I and R individuals and of detection of R individuals were very robust to increasing state uncertainty. In contrast, the probabilities of survival and detection of S individuals, and the infection and recovery probabilities showed high biases and low precisions when state uncertainty was >50%, particularly when the assignment of the S state was reduced. Considering this specific disease scenario, SIR-MECMR models are globally robust to state uncertainty and heterogeneity in state assignment, but the previously mentioned parameter estimates should be carefully interpreted if the proportion of U is high.

## Introduction

Describing the dynamics of infectious diseases and accurately quantifying their effects on hosts is of critical relevance for human public health and the associated economic costs. Infectious diseases of wildlife threaten humans or livestock, either as direct zoonosis, through contact with or ingestion of infected animals [e.g., rabies: (1); Ebola: (2); brucellosis: (3)], through air-borne infectious particles [e.g., avian influenza: (4)], or via other wildlife species that act as intermediate hosts or reservoirs [e.g., West-Nile fever: (5)]. Infectious diseases can also seriously reduce the population size of endangered wildlife species [e.g., fungal infection chytridiomycosis in frogs and salamanders: (6); white-nose syndrome in bats: (7); facial tumor disease in the Tasmanian devil: (8)]. They are now recognized as a major and urgent issue in the context of global biodiversity loss (9, 10).

To determine the impact of a disease on key demographic parameters such as individual survival or to investigate temporal variation in disease exposure, researchers need to monitor the health status and fate of individuals. Observation of clinical signs and collection of biological samples (to assess seroprevalence or screen for the presence of pathogens) are common diagnostic approaches, which separately or in combination may be used to determine the health status of free-ranging animals (11–15). Even so, individually known animals may be (1) undetected during a field session, or detected but their health status may be (2) unknown e.g., when no biological sample could be collected, or (3) uncertain e.g., when clinical signs are subtle and similar for different pathogens, or when diagnostic tests include false negative or false positive results (16). These situations regularly occur when the model species is elusive, or when biological samples can only be collected opportunistically or by non-invasive techniques (15).

Individuals with such “imperfect” information have traditionally been taken out from data sets and statistical analyses. Not only does this reduce sample size and hence statistical power, it can also increase bias and/or reduce precision in the estimates of state-specific demographic (17) or eco-epidemiological parameters (18). Substantial improvements in addressing these issues have been made possible by the development and application of multi-state hidden Markov models, also termed multi-state capture-mark-recapture (CMR) models (19). Along the same lines, multi**-**event CMR (MECMR) approaches have recently been developed (20, 21) and applied to improve the estimates of key demographic or eco-epidemiological parameters in population ecology (17, 22) and disease ecology (18). These models, when applied to epidemiological data, consider discrete infection states such as “susceptible” (S), “infected” (I) or “recovered” (R), and reduce bias/increase precision in the estimates of state-specific parameters (as opposed to multi-state CMR models) by accounting for several processes. First, they account for “imperfect detection” of individuals, which can occur when some individuals in the population are not observed during a field visit. Second, they account for “infection state uncertainty”, also termed “partial observation”, which can occur when individuals in the population are observed but their infection state could not be determined (12, 15, 18, 23–25). Finally, they also account for “infection state misclassification”, which can occur when some individuals are observed as alive and their infection state could only be assigned with some uncertainty. For instance, the state S may have been assigned although there was a risk that the individual was in fact I or R (12, 16, 26). MECMR models provide a powerful methodology to estimate state-transition and apparent survival parameters even under such circumstances (20, 21). However, their performance in terms of bias and precision of parameter estimates when uncertainty increases importantly, has not yet been investigated, to our knowledge. The current study aimed to develop a modeling framework to address this issue. For this purpose, we used simulations and focused on infection state uncertainty (i.e., partial observation), when some individuals are detected but not sampled during a field session and as a result are assigned an unknown (“U”) infection state on a given occasion.

**Figure 1:**
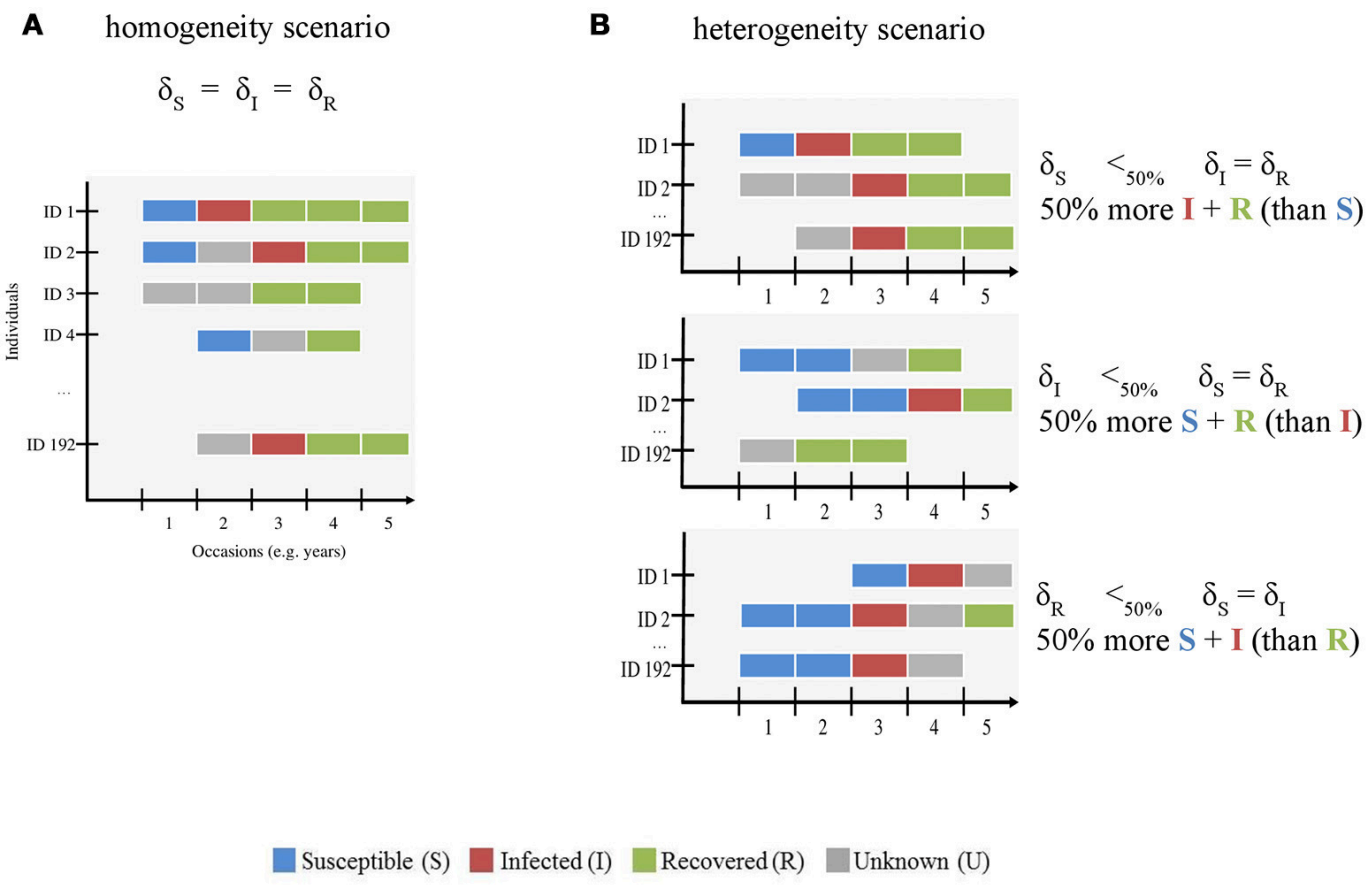
Schematic representation of the simulated CMR data sets with **(A)** homogenous and **(B)** heterogeneous infection state uncertainty. The x-axis shows the number of occasions (here 5, see Table 1) and the y-axis shows the individuals (ID, here 192 individuals were simulated). This figure is meant to visually distinguish between both modeled scenarios. The gradient of uncertainty is not shown, for simplicity. For both scenarios, we used the assignment probabilities of susceptible (S) (δ_S_), infected (I) (δ_I_), and recovered (R) (δ_R_) states to simulate variation in infection state uncertainty. S, I, R and unknown (U) states are shown in blue, red, green, and gray, respectively. In the homogeneity scenario **(A)**, we gradually increased uncertainty by decreasing the probability of assigning each infection state by an equal amount in the simulated data set. In the heterogeneity scenario **(B)**, we varied the assignment probability of all states across a gradient as for **(A)** and reduced the value of one state by 50% compared to the other two. For instance, in the first case (right panel, top figure), the number of S states (blue) is lower than that of I (red) and R (green) states.

First, we evaluated how increasing infection state uncertainty (i.e., increasing the proportion of U individuals) affected bias and precision of eco-epidemiological parameter estimates. This knowledge is essential to assess the reliability of MECMR approaches when the percentage of reliably sampled individuals in a free-ranging population is low, and therefore data on infection states are scarce. We gradually increased uncertainty by decreasing the probability of assigning each infection state by an equal amount in the simulated data set (“homogeneity” scenario, Figure 1A). We expected bias and precision to increase and decrease, respectively, with increasing state uncertainty.

Second, we asked how heterogeneity in the probability of assigning infection states influenced bias and precision (“heterogeneity” scenario, Figure 1B) when infection state uncertainty increased. The assignment of infection states may depend on several diagnostic methods such as field observations of clinical signs and/or laboratory techniques including serology and/or molecular screening techniques [e.g., (11, 15)]. These diagnostic methods are often specific to the assignment of given infection state(s). For instance, serological tests such as antigen ELISAs are typically used to determine S and R states on the basis of the concentration thresholds of antibody titres (1, 12, 26, 27) and clinical signs and RT-PCR results are often used to determine the I state [e.g., (11, 15, 28, 29)]. In habituated free-ranging populations, where observations of known individuals from a close distance are feasible, clinical signs for some infectious and virulent diseases can be conspicuous [e.g., Ross River virus, (30)]. If these populations are monitored by non-invasive techniques, the availability of sera for measuring antibody titres would typically be limited. In this case, the probabilities of detecting animals in S, I and R states might be similar, but the probabilities of assigning the correct infection state to each detected animal may differ—-with the assignment of the I state being more likely than that of the S or R states. Other scenarios are possible where the assignment of the I state may be lower than that of the S and R states. When immune processes control infection, the period in which an infected animal sheds viral particles will be limited, thereby restricting the time-window in which genetic screening will identify the infection. Furthermore, for viral diseases that have periods of subclinical infection, such as canine distemper virus [CDV, e.g., (15)], or latent infection such as equine herpesvirus (31), when virus particles are not shed at all, genetic screening methods may not correctly identify infected individuals. This may result in a low assignment of the I state. It is unclear how such heterogeneity in the assignment of true infection states affects bias and precision in model parameter estimates, in particular when infection state uncertainty is high.

Answering both questions would be very useful to optimize study designs and disease surveillance programs (32). For both scenarios, we fitted SIR-MECMR models with a fictive set of parameter values as input to simulate data, and investigated bias and precision for different levels of uncertainty. We simulated the case of a virulent disease inducing life-long immunity.

## Methods

### Model structure

We built a multi-event capture-mark-recapture (MECMR) model (20) depicting Susceptible (S)—Infected (I)—Recovered (R) dynamics to estimate survival, infection and recovery probability while accounting for imperfect detection and infection state uncertainty (21). The model had three infection states (S, I, and R) plus the dead state (D), and five underlying events—not detected, detected and diagnosed as S, detected and diagnosed as I, detected and diagnosed as R, detected and undiagnosed (i.e., set as unknown: U). The model included the following state-dependent parameters: survival probability (*ϕ*), infection probability (*β*) (i.e., transition from S to I state), recovery probability (*γ*) (i.e. transition from I to R), detection probability (*p*), and assignment probability of infection states (δ).

**Figure 2:**
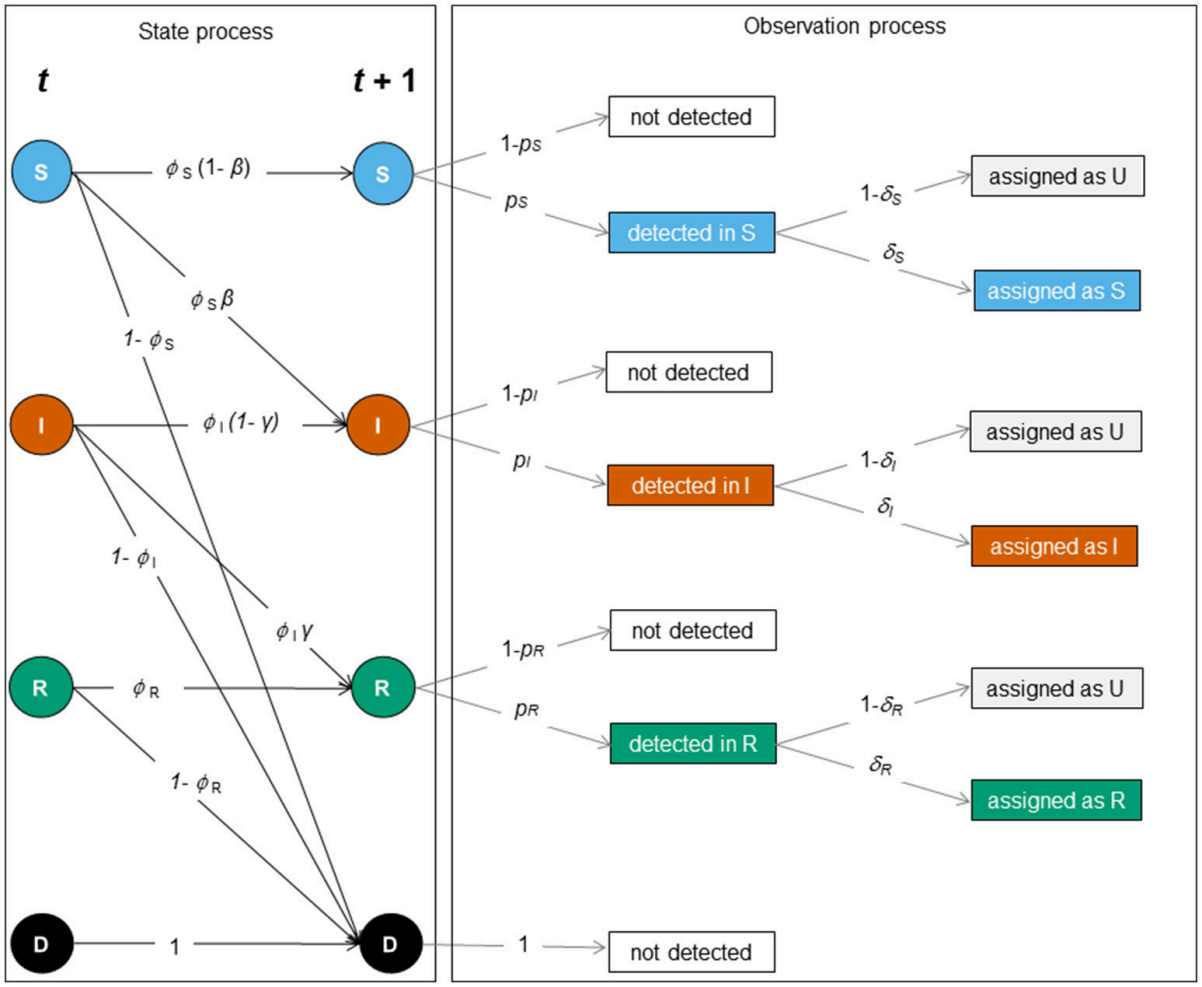
Schematic representation of the SIR dynamics with state uncertainty between two sampling occasions, *t* and *t* + 1. The state or biological process (left) and the observation process (right) underlie the construction of the MECMR model. In the state process [corresponding to the matrix *SIR_Model*, Equation (1)], solid circles indicate infection states (S [blue]: “susceptible”, I [orange]: “infected,” R [green]: “recovered”) and the dead state (D, [black]). The solid black arrows indicate transition probabilities between those states as a function of the probability of surviving in a given state (*ϕ*_*i*_, with *i* specific for S, I and R), i.e. the probability of staying susceptible (1 – *β*), becoming infected (*β*), staying infected (1- *γ*) or recovering (*γ*). The observation process was composed of two steps: detection of individuals [corresponding to the matrix *Obs1*, Equation (2)], and assignment of infection states [corresponding to the matrix *Obs2*, Equation (3)]. Events are shown in black solid boxes. Solid gray arrows indicate the detection probability of individuals in a given state (*p*_*j*_ with *j* being specific for S, I, and R) and the assignment probability of individuals in a given state (δ_*k*_ with *k* being specific for S, I and R).

The construction of MECMR models requires the formulation of two processes: (1) the biological process (or state process), which accounts for transitions between the three infection states, conditional on the survival of individuals in their given state, and (2) the observation process, which accounts for imperfect detection of individuals and state uncertainty. The model's likelihood is a function of these two processes (20, 21) that can, in turn, be represented as matrix blocks, as shown below and in Figure 2.

The matrix for the biological process (*SIR_Model*) was the product of two matrices representing transitions between infection states and survival and was as follows:

$$SIR\_Model=\begin{array}{c} \;\begin{array}{cccc} S\; & I & \;R & \;D \end{array}\\ \begin{array}{cc} \begin{array}{c} S\\ I\\ R\\ D \end{array} & \left[\begin{array}{cccc} \phi S*(1-\beta ) & \phi S*\beta & 0 & 1-\phi S\\ 0 & \phi I*(1-\gamma )\; & \phi I*\gamma & 1-\phi I\\ 0 & 0 & \phi R & 1-\phi R\\ 0 & 0 & 0 & 1 \end{array}\right] \end{array} \end{array}$$

with ϕ_*S*_, *ϕ*_*I*_ and *ϕ*_*R*_ the survival probability of individuals in S, I and R states, respectively, *β* the infection probability, *1-* *β* its complement, *γ* the recovery probability, and 1- *γ* its complement. Each entry in the matrix *SIR_Model* is the probability of transition from a “starting” infection state (4 rows corresponding to the infection states S, I, R, and the dead state D) to a “subsequent” infection state (4 columns corresponding to S, I, R, and D), conditional on the survival of individuals in their given state.

The matrix accounting for imperfect detection in the observation process (*Obs1*) was as follows:

$$Obs1=\begin{array}{c} \;\begin{array}{cccc} nd\; & d^{S} & d^{I} & \;d^{R} \end{array}\\ \begin{array}{cc} \begin{array}{c} S\\ I\\ R\\ D \end{array} & \left[\begin{array}{cccc} 1-pS & pS & 0 & 0\\ 1-pI & 0 & pI & 0\\ 1-pR & 0 & 0 & pR\\ 1 & 0 & 0 & 0 \end{array}\right] \end{array} \end{array}$$

with *p*_*S*_, *p*_*I*_, and *p*_*R*_ the detection probability of S, I and R individuals, respectively, and *1- p*_*S*_*, 1- p*_*I*_, and *1- p*_*R*_ their complements. Each entry in the matrix *Obs1* is the probability of being detected in a given infection state. The 4 rows correspond to the infection states S, I, R, and the dead state D and the 4 columns correspond to the following events: not detected (nd), individual detected in state S (d^S^), detected in state I (d^I^) and detected in state R (d^R^).

The matrix accounting for the infection state assignment process (*Obs2*) was as follows:

$$Obs2=\begin{array}{c} \;\begin{array}{cccc} nd\;a^{S}\; & a^{I} & a^{R} & a^{U} \end{array}\;\\ \begin{array}{cc} \begin{array}{c} nd\\ a^{S}\\ a^{I}\\ a^{R} \end{array} & \left[\begin{array}{cccc} 1 & 0 & 0 & 0\\ 0 & \delta S & 0 & 0\\ 0 & 0 & \delta I & 0\\ 0 & 0 & 0 & \delta R \end{array}\begin{array}{c} 0\\ 1-\delta S\\ 1-\delta I\\ 1-\delta R \end{array}\right] \end{array} \end{array}$$

with δ_*S*_, δ_*I*_, and δ_*R*_ the assignment probability of individuals to S, I, and R states, respectively, and *1-* δ_*S*_*, 1-* δ_*I*_, and *1-* δ_*R*_ their complements. Each entry in the matrix *Obs2* is the probability of being assigned to a given infection state. The 4 rows, equivalent to the columns of the matrix accounting for imperfect detection *Obs1* correspond to the following events: not detected (nd), detected in state S (d^S^), detected in state I (d^I^) and detected in state R (d^R^). The 5 columns correspond to the following events: not detected (nd), detected and assigned as a S (a^S^), detected and assigned as an I (a^I^), detected and assigned as a R (a^R^), detected and assigned as a U (a^U^).

The product of the detection matrix (Obs1) and the state assignment process (Obs2) then represents the observation process (Figure 2). State uncertainty was homogeneous when δ_*S*_ = δ_*I*_ = δ_*R*_ and heterogeneous when δ_*S*_ ≠ δ_*I*_ and δ_*R*_, or δ_*I*_ ≠ δ_*S*_ and δ_*R*_, or δ_*R*_ ≠ δ_*S*_ and δ_*I*_. Note that the MECMR model becomes a multi-state CMR model when δ_*S*_ = δ_*I*_ = δ_*R*_ = 1.

### Simulations

#### Data sets and input parameter values

For all analyses, we considered variation in the level of uncertainty ranging between 20% and 90% and increased this level by 10% at each iteration. We set the number of occasions, corresponding for instance to years of observation, equal to five to mimic the conditions of most eco-epidemiological CMR studies, which are typically based on a few years of data collection during a disease outbreak [e.g. (11, 25)]. For each level of uncertainty, we simulated 1000 data sets. For this, we first simulated the “true states” of individuals by applying for each individual the survival and infection processes captured by the matrix of the biological process *SIR_Model* (1) from the first detection occasion until the last one. Second, we considered the encounter histories of individuals by applying the observation process described by the matrices *Obs1* (2) and *Obs2* (3), for each individual alive along the sampling period. These three matrices were implemented with input values for the following parameters: initial state probabilities (π), the survival probability (*ϕ*) of infection states, the infection probability (*β*), the recovery probability (*γ*), the detection probability (*p*) of infection states, and the assignment probability of infection states (δ).

Input parameter values are presented in Table 1. We mimicked the case of a virulent disease, which induces lifelong-immunity, i.e., characterized by a high infection probability, a low survival probability once infected and a low recovery probability. The number of new released individuals (i.e., previously unmarked), at each occasion, in each state was 12, 24, and 12, for S, I, and R respectively, to simulate a case where the disease already started spreading at the beginning of the study. Hence, initial state probabilities of S, I and R states were as follows: π_*S*_ = 0.33, π_*I*_ = 0.66, π_*R*_ = 0.33. We chose this set of input parameter values because we expected bias to be higher and precision to be lower than in less virulent disease scenarios, as suggested by our preliminary runs (not shown).

**Table 1:** Fictive input parameter values for the probabilities of surviving (*ϕ*), becoming infected (*β*), recovering (*γ*) and of detection (*p*), which were used to simulate data sets.

**Parameter**	**Description**	**Estimate**
*ϕ*_*S*_	Survival probability of susceptible	0.90
*ϕ_*I*_*	Survival probability of infected	0.50
*ϕ_*R*_*	Survival probability of recovered	0.90
*β*	Infection probability	0.90
*γ*	Recovery probability	0.30
*p*	Detection probability	0.50

*We mimic the case of a virulent disease (i.e., high* β, *low ϕ_I_, low* γ), *which induces lifelong immunity, in a population where animals have a moderate detection probability*.

#### Definitions of bias and precision

At the end of the procedure described above, and for each level of uncertainty ranging between 20% and 90%, we quantified bias and precision of parameter estimates. Biases were the differences between the average of parameter estimates across the 1000 simulations and the “true values” of the parameters (i.e., the input parameter values shown in Table 1). To assess precision, we calculated the mean squared error (MSE) as the mean of the squared differences between simulation outcomes and true values.

#### Homogeneity scenario

To determine the impact of an increasing level of state uncertainty (from 20 to 90%) on bias and precision, we progressively decreased the assignment probability of S, I and R states by identical values (homogeneity scenario, Figure 1A), from 0.8 down to 0.1

#### Heterogeneity scenario

To determine the impact of heterogeneity in the assignment probability of infection states on bias and precision when state uncertainty increases, we progressively decreased the assignment probability of S, I and R states from 0.8 down to 0.1 to simulate as above an increasing level of state uncertainty from 20 to 90%, but reduced by 50% the value of assignment probability for one state (e.g., for S) while keeping the two other assignment probabilities (e.g., for I and R) similar. We repeated this procedure for all three states (heterogeneity scenario, Figure 1B).

#### R programming code

We used R.3.5.0. (33) for all analyses. We used the R package “TMB” 1.7.13 (34) to fit MECMR models to simulated data and perform maximum likelihood estimation. This study is the first application of the TMB package to MECMR models to our knowledge. Because in this package models are directly formulated in C++, TMB speeded up the optimization process substantially, making the analyses 100 times faster than the native R code based on a benchmarking analysis.

### Case study

In the Supplementary Material Table S2 we present a similar analysis as the one described above, where parameter estimates originate from a MECMR model developed for and fitted to 20 years of data on CDV infection in the spotted hyena (*Crocuta crocuta*) population in the Serengeti National Park, in Tanzania (15). We illustrate using this case study that our simulation framework can easily be applied to real data.

## Results

### Overview

**Figure 3:**
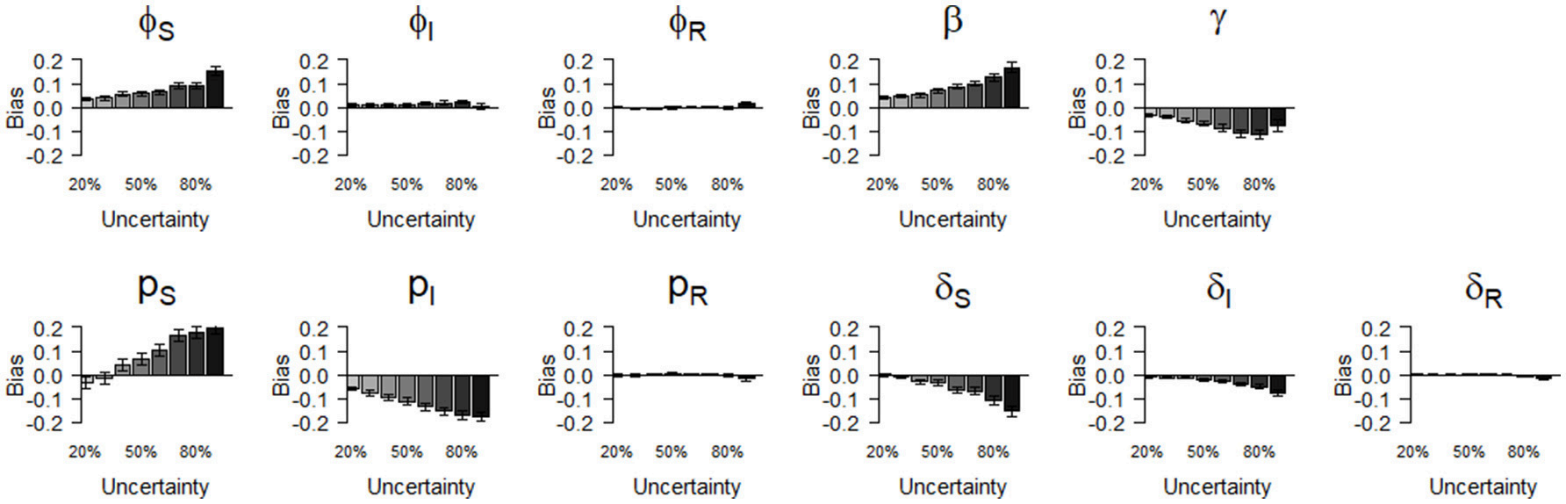
Bias (mean difference between the parameter value estimated via simulations and the input parameter value) of parameter estimates as a function of homogeneously increasing infection state uncertainty. Error bars represent 95% CI. The increasing state uncertainty on the x-axis corresponds to a decreasing probability of assigning an infection state. We used the following notations for the parameters: *ϕ*_*S*_, *ϕ*_*I*_, and *ϕ*_*R*_ for the survival probability of individuals in S, I, and R states, *β* for the infection probability, *p*_*S*_, *p*_*I*_ and *p*_*R*_ for the detection probability and δ_*S*_, δ_*I*_ and δ_*R*_ for the assignment probability of individuals in S, I, and R states, respectively.

**Figure 4:**
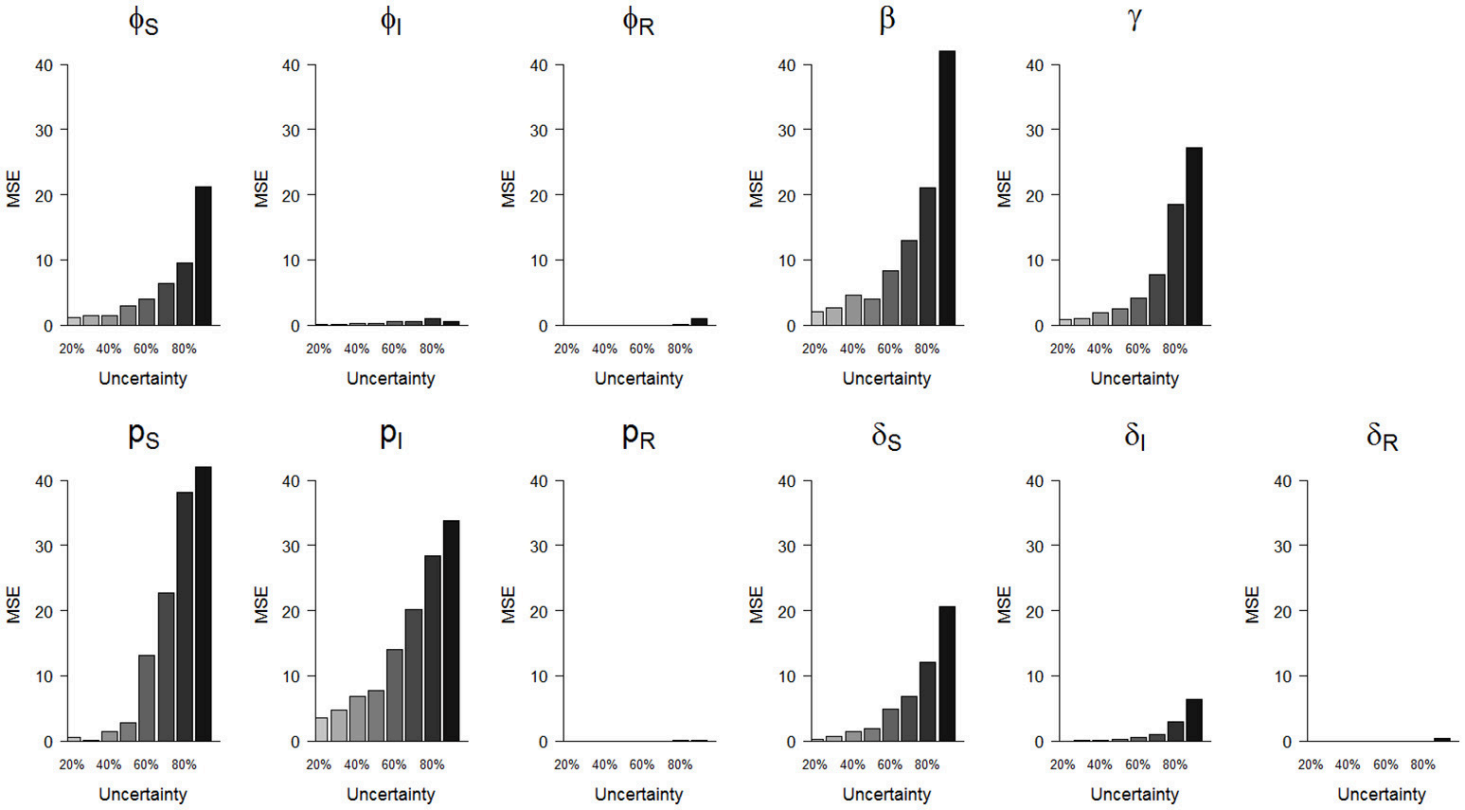
Precision (mean squared error, MSE)) of parameter estimates as a function of homogeneously increasing infection state uncertainty. The increasing state uncertainty on the x-axis corresponds to a decreasing probability of assigning an infection state. We used the following notations for the parameters: *ϕ*_*S*_, *ϕ*_*I*_ and *ϕ*_*R*_ for the survival probability of individuals in S, I and R states, *β* for the infection probability, *p*_*S*_, *p*_*I*_, and *p*_*R*_ for the detection probability and δ_*S*_, δ_*I*_, and δ_*R*_ for the assignment probability of individuals in S, I and R states, respectively.

**Figure 5:**
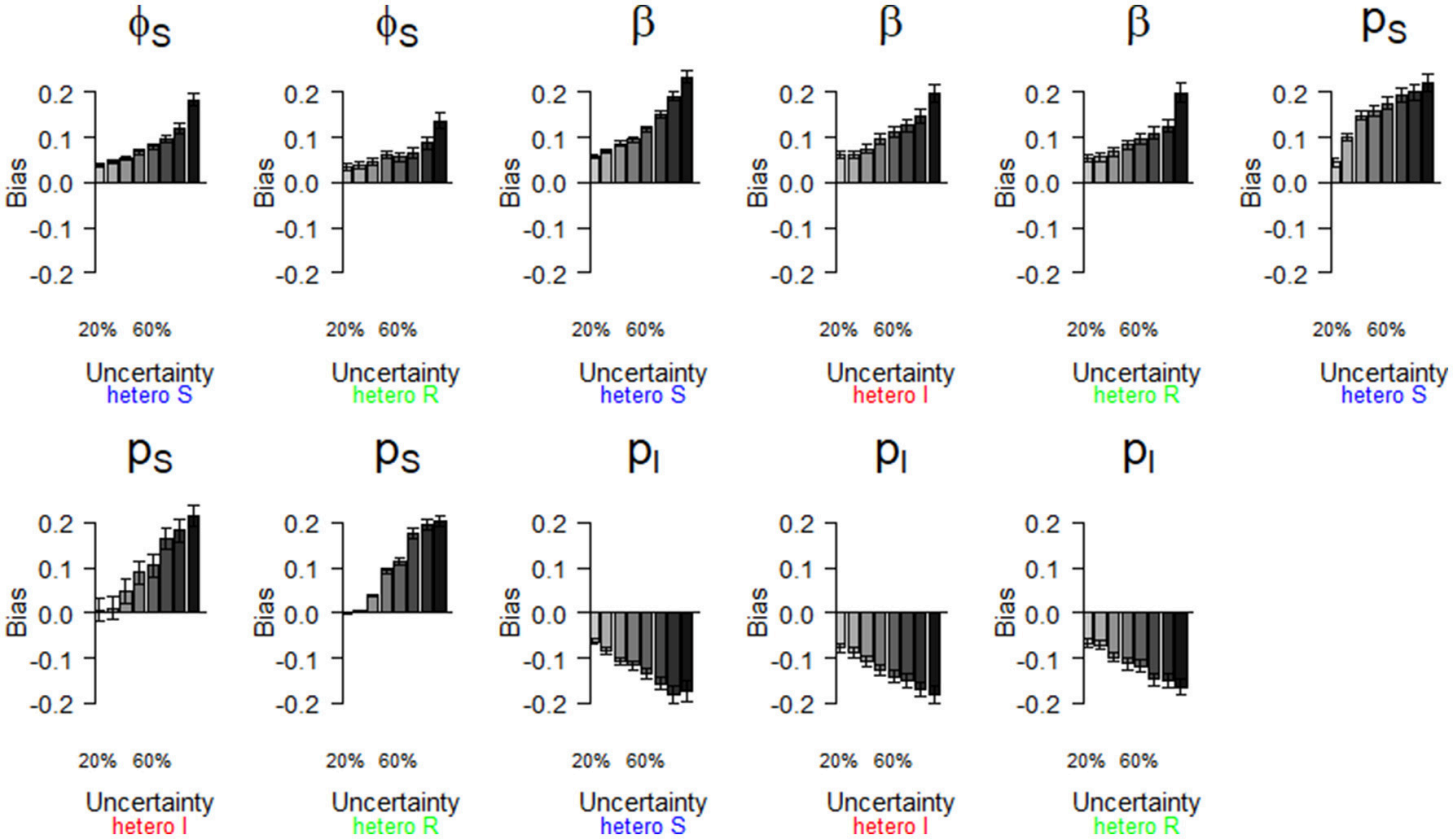
Parameter estimates with a bias greater than |0.2| as a function of a heterogeneous increase in state uncertainty. Error bars represent 95% CI. The increasing state uncertainty corresponds to a decreasing probability of assigning an infection state. We used the following notations for the parameters: *ϕ*_*S*_ for the survival probability of individuals in the S state, *β* for the infection probability, *p*_*S*_ and *p*_*I*_ for the detection probability of individuals in S and I states, respectively. Scenarios of assignment heterogeneity were reducing by 50% the assignment probability of the S state compared to the assignment probability of the I and R states (“hetero S” (blue)), reducing by 50% the assignment probability of the I state compared to the S and R states (“hetero I” (red)) and reducing by 50% the assignment probability of the R state compared to S and I states (“hetero R” (green)).

**Figure 6:**
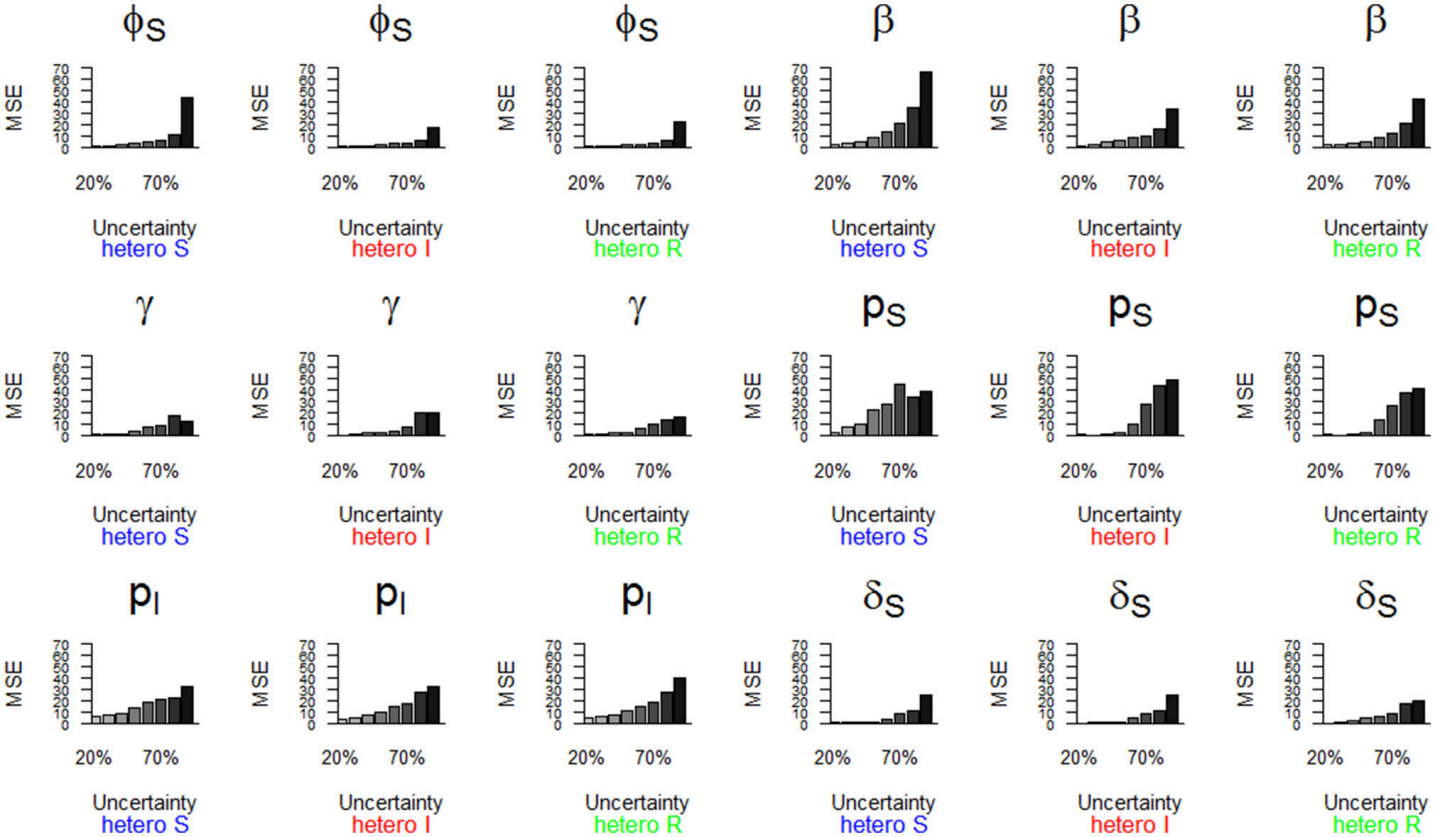
Parameter estimates with a precision (MSE) greater than 15 as a function of a heterogeneous increase in state uncertainty. The increasing state uncertainty corresponds to a decreasing probability of assigning an infection state. We used the following notations for the parameters: *ϕ*_*S*_ for the survival probability of individuals in the S state, *β* for the infection probability, *γ* for the recovery probability, *p*_*S*_ and *p*_*I*_ for the detection probability of individuals in S and I states, and δ_*S*_ for the assignment probability of individuals in the S state. Scenarios of assignment heterogeneity were reducing by 50% the assignment probability of the S state compared to the assignment probability of the I and R states (“hetero S” (blue)), reducing by 50% the assignment probability of the I state compared to the S and R states (“hetero I” (red)) and reducing by 50% the assignment probability of the R state compared to S and I states (“hetero R” (green)).

When state uncertainty increased from 20% to 90%, bias tended to increase (in the positive or negative range) for most parameter estimates, irrespective of whether the increase was homogeneous across infection states (Table 2, Figure 3) or heterogeneous (Table 2, Figure 5). Similarly, precision tended to decrease with increasing state uncertainty (see Table 2 and Figure 4 for homogeneous state uncertainty and Figure 6 for heterogeneous state uncertainty). All values for bias and precision are provided in Table S1.

**Table 2:**
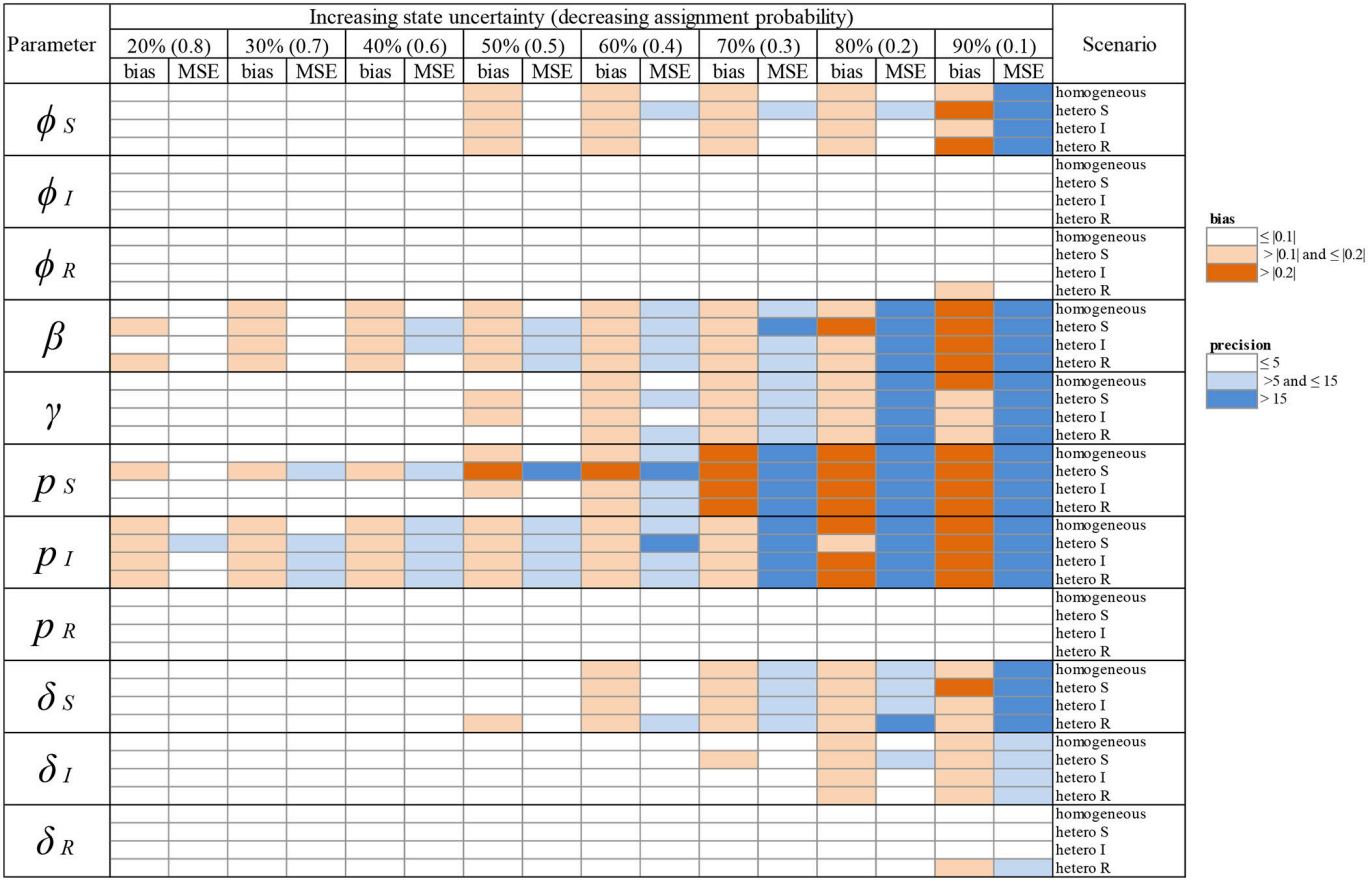
Overview of variation in bias (mean difference between the parameter value estimated via simulations and the input parameter value) and precision (minimum squared error, MSE) of parameter estimates in relation to an increasing infection state uncertainty (ranging between 20 and 90%) implemented as a decreasing assignment probability of infection states (ranging between 0.8 and 0.1). Bias and precision were calculated in data sets simulated under different scenarios of assigning infection states: homogeneous or heterogeneous assignment probabilities, in which the assignment of S (“hetero S”), I (“hetero I”) or R (“hetero R”) states was reduced by 50% in comparison to the two other infection states. We used the following notations for the parameters: *ϕ*_*S*_, *ϕ*_*I*_ and *ϕ*_*R*_ for the survival probability of individuals in S, I, and R states, *β* for the infection probability, *γ* for the recovery probability, *p*_*S*_, *p*_*I*_ and *p*_*R*_ for the detection probability and δ_*S*_, δ_*I*_ and δ_*R*_ for the assignment probability of individuals in S, I, and R states, respectively. Orange: cases where bias (in absolute value) was >0.1 and ≤ 0.2 (light orange) and > 0.2 (dark orange). Blue: cases where precision was > 5 and ≤ 15 (light blue) and > 15 (dark blue). For simplicity reasons we chose to show these broad categories here and to present the values for bias and precision in the Supplementary Material Table S1.

### Homogeneity scenario

We show (Figure 3) how bias varied with increasing state uncertainty when we homogeneously decreased the assignment probability of infection states. The maximal bias (in absolute values) was 0.2; for the probabilities of infection (*β*), recovery (*γ*), the detection of S (*p*_*S*_) and I (*p*_*I*_), estimated between 50 and 90 % level of uncertainty (Figure 3, Table S1). For the probabilities of survival of I (φ_*I*_) and of R *(*φ_*R*_), the detection of R (*p*_*R*_) and the assignment of R *(*δ_*R*_), bias was below 0.05 in absolute value, even when state uncertainty was at a maximum (90%) (Table 2, Figure 3).

In terms of precision, the probabilities of infection (*β*) and of detection of S (*p*_*S*_) were the most sensitive parameters to increasing state uncertainty, reaching an MSE value of 42.0 when state uncertainty was 90% (Figure 4, Table S1). This was followed by the detection probability of I (*p*_*I*_) and the recovery probability (*γ*), which reached MSE values of 33.8 and 27.3, respectively, when state uncertainty was 90% (Figure 4, Table S1). Similar to the results for bias, the precision of the probabilities of survival of I (*ϕ*_*I*_) and of R *(ϕ*_*R*_), the detection of R (*p*_*R*_) and the assignment of R *(*δ_*R*_) remained high even when state uncertainty was very high, as none of these parameter estimates reached an MSE value strictly higher than 1 (Table 2, Figure 4).

### Heterogeneity scenario

We show (Figure 5) which parameter estimates had a high bias (i.e., >|0.2|, see Table 2) when state uncertainty was heterogeneously increased among infection states. All scenarios (i.e., “hetero S”, “hetero I,” “hetero R”, see Table 2) resulted in high biases under some conditions (Figure 5). Globally, bias increased progressively for all parameter estimates shown in Figure 5, when state uncertainty increased. For the survival of S (*ϕ*_*S*_), the ranges of bias values and the patterns of bias increase were similar for two scenarios (“hetero S” and “hetero R”). For the probability of infection (*β*), the increase in bias with increasing state uncertainty was more pronounced for the “hetero S” scenario than for the two others. Interestingly, bias for this parameter estimate and for the detection of I (*p*_*I*_) was at least >|0.1| (Table 2) even when state uncertainty was low (i.e., 20%). All parameters for the biological process, except the recovery probability (*γ*) (see Table S1) showed a tendency to being overestimated when state uncertainty increased. In contrast, the recovery probability showed a tendency to being underestimated when state uncertainty increased.

For the observation process, the detection probability of S (*p*_*S*_) showed an increase in bias with increasing state uncertainty that was more pronounced for the “hetero S” scenario than for the two others (Figure 5, Table 2). For the detection probability of I (*p*_*I*_), the response to increase in state uncertainty, in terms of bias, was similar for all three scenarios (Figure 5, Table 2). In all scenarios, the probability of detection of R (*p*_*R*_) was only biased minimally (Table 2).

The parameter estimates with the highest bias, as shown in Figure 5, also had the lowest precision (i.e., MSE >15) (Table 2, Figure 6). In addition, the recovery probability (*γ*) showed a moderate increase in its MSE value in all scenarios when state uncertainty increased. The probability of infection (*β*) reached the highest MSE value (i.e., 66.8) in the “hetero S” scenario. For the probability of survival (*ϕ*_*S*_) and the probability of infection (*β*) the decrease in precision was more important in the “hetero S” scenario than in the two others. Interestingly, MSE showed a marked increase when state uncertainty reached 90% for the survival probability of S (*ϕ*_*S*_) and the infection probability (*β*), in all scenarios.

Overall, the scenario “hetero S” resulted in more parameters being importantly over- or underestimated and imprecise when state uncertainty increased (Table 2) than the scenarios “hetero I” and “hetero R”.

## Discussion

Uncertainty about the health status of animals is inherent to field studies of free-ranging wildlife populations exposed to naturally occurring pathogens (18, 35). Whereas it is known that MECMR models can deal with various types of uncertainty in the data, previously it was unclear how much infection state uncertainty could be tolerated to provide reliable estimates of eco-epidemiological parameters. Here, we tested the robustness of these models in terms of bias and precision of parameter estimates when uncertainty about the health status of individuals in the monitored population increases, mimicking the specific and extreme case of a virulent disease that induces lifelong immunity. For most parameter estimates and in all scenarios, we found a progressive increase in bias and a progressive decline in precision with increasing state uncertainty. This was expected, even if in some cases this increase/decline was minimal (Table 2, Figures 3–6). The results from our simulations indicate that overall these models are robust in terms of bias to variation in infection state uncertainty because even at a very high level of uncertainty in the data set (90%), maximum bias in absolute value was moderate (0.3 for the probability of infection (*β*)). The loss in precision was more important than the increase in bias when state uncertainty increased, as the MSE values were high when state uncertainty reached 90% (the highest value being 66.8 for the probability of infection (*β*)).

### Assignment probability and proportion of unknown

Here we used the assignment probability of infection states to model the proportion of unknown infection states in the data set, rather than modifying the proportion itself in the data set. This allowed us to know exactly which parameter values were used as input. We have verified that there was a direct and obvious correlation between the assignment probability of infection states and the proportion of individuals in an unknown state in the data set (results not shown).

### Impact of S, I, and R states (SIR dynamics) on bias and precision

The outcomes from all modeling scenarios indicated that for the probabilities of survival, detection and assignment of infection states, the infection state itself had a critical impact on bias and precision of parameter estimates. The results shown in Table 2 indeed indicate that the bias was highest (and the precision lowest) for all parameter estimates related to the S state, then followed by those related to the I state. In contrast, all parameter estimates linked to the R state showed very little bias (i.e., ≤ |0.10|) and high precision (i.e., MSE ≤5), irrespective of the modeling scenario.

These results are a likely consequence of the fact that we modeled disease dynamics under the assumption that the pathogen induces lifelong immunity—i.e., SIR dynamics where individuals in the R state can never become S again (Figure 2). This illustrates for instance the case of morbilliviruses such as CDV (15), measles, or rinderpest (36), classical swine fever (11) or rabbit haemorrhagic disease (25). For such types of diseases, the final data sets should comprise far more individuals in the R state—a “final” state, than in the S or the I states. This pattern should be influenced by the duration of the study and whether or not the duration of the study matches the duration of the disease epidemic. If the duration of the study extends well beyond the duration of the epidemic [as in e.g., (15)], then this may leave enough time for the pool of S to rebuild, and this pattern may be attenuated. In contrast, this pattern of a lower bias and higher precision for parameter estimates linked to the R state may be particularly apparent when studies are based on just a few years of data collection during a disease outbreak [e.g., (11, 25)].

In our fictive data sets, this effect may have been further exacerbated by the fact that we used a very high infection probability value as input (*β* = 0.90, see Table 1), which resulted in the rapid spread of the disease in the population and a relatively rapid accumulation of individuals in the R state. It would be interesting to assess if this pattern is also observed for the I state in SI disease dynamics where the transition from the I state to the S state is not possible, as for tuberculosis in badgers (16). Similarly, we would expect the pattern to be absent for other types of modeled disease dynamics such as avian influenza (4), where infection does not provide lifelong immunity upon recovery from infection, and individuals can become S again (e.g., SIS or SEIS models). Our flexible code allows to model these other types of disease dynamics.

### Heterogeneity in assigning S, I, and R states

Irrespective of any variation in detection probabilities among infection states, it is likely that infection states will not have equal probabilities of being assigned in field studies [e.g., (1, 15)]. Such heterogeneity may result from the manifestation of the disease in the host and/or the diagnostic method employed. For instance, Faustino et al. (28) observed house finches infected with conjunctivitis and assigned them to either an infected or not infected state, based on the observation of clinical signs of the disease manifested around the bird's eyes. When (18) reanalyzed this data set by applying a MECMR model, they found that the assignment probability varied with infection state. The difficulty of assigning the infection status of birds observed from a long distance resulted in a lower assignment of the infected state than the non-infected state (irrespective of the detection probability).

When infection states are assigned based on the outcome of one diagnostic test such as “seropositive” and “seronegative”, as for free-ranging rabbits exposed to rabbit haemorrhagic disease (25) or bisons exposed to brucellosis (3), we expect heterogeneity in the assignment of infection states to be minimized as compared to cases where more than one diagnostic method is employed [e.g., (11, 15)]. This is because the assignment of infection states in such cases depends on the performance of only one type of diagnostic test (e.g., ELISAs to measure antibody titres) rather than several, which should minimize bias introduced by the diagnostic approach itself.

To our knowledge, our study is the first to investigate the impact of such heterogeneity on bias and precision of capture-recapture eco-epidemiological parameter estimates. We did not find any striking differences between cases where state uncertainty varied homogeneously or heterogeneously (Table 2). However, as mentioned previously, we found that the scenario “hetero S”, in which the assignment probability of the S state was half of that of the two others, tended to result in a higher bias and a lower precision of parameter estimates. It is probable that the pattern linked to the SIR dynamics described above is further exacerbated when the assignment probability of the S state is lower than that of the I and R states. This suggests that for SIR diseases and other types of diseases where individuals accumulate in a “final” infection state, researchers should attempt whenever possible to maximize the assignment probability of the S state (or any other “starting” state), e.g., by increasing sampling effort to collect serum, or at least be aware and mention that these specific parameter estimates may be biased and imprecise when state uncertainty is high.

### Impact of our set of input parameter values

In this study, we chose to simulate an “extreme” disease case, characterized by a virulent pathogen inducing life-long immunity, and high and low probabilities of infection and recovery, respectively, such as Ebola in western lowland gorillas (37) or highly pathogenic avian influenza viruses of the H5N1 subtype in humans (38). Our preliminary model runs indicated that bias and precision of parameter estimates would be more affected by state uncertainty in this situation than in “milder” disease scenarios. However, it is possible that our conclusions on the relatively high robustness of eco-epidemiological parameter estimates in presence of high levels of state uncertainty are specific to the set of input parameter values we used. A future interesting step would thus be to conduct a sensitivity analysis to assess the importance of key input parameters such as the probabilities of infection and recovery, survival of infected individuals or the detection of S,I, and R. For instance, these probabilities could be varied by 1% within a given range (e.g., 0.1 and 1), while maintaining the other parameter estimates constant. Bias and precision of all parameter estimates could then be estimated at each iteration, for each level of uncertainty. Other critical parameters may be the number of occasions and/or the initial probabilities. We chose here a relatively short time scale (5 occasions) to mimic the conditions of most eco-epidemiological CMR studies, typically based on a few years of data collection during a disease outbreak [e.g., (11, 25)]. We show in the Supplementary Information 2 that our framework can well be applied to other timescales, such as in longitudinal long-term research projects, as we used there a far larger number of occasions (20 years) [e.g., spotted hyenas infected with CDV in (15)]. All else being equal, we generally expect that bias and precision of parameter estimates should decrease when the number of occasions increases. Our framework is highly flexible, the type of disease dynamics modeled can be modified easily, and any given set of input parameter values can be tested to assess bias and precision of eco-epidemiological parameter estimates.

### State uncertainty: partial observation and state misclassification

In this study, we chose to focus on “partial observation” [of infection states], which occurs when individuals in the population are observed (alive) but their infection state could not be determined (12, 15, 18, 23–25). We hence deliberately ignored “state misclassification”, the other component of the concept of state uncertainty, which occurs when some individuals are observed (alive) and their infection state could be assigned, but with some (unmeasured level of) uncertainty. Although state misclassification is in fact likely to be widespread, due to false positive and false negative test results, this type of uncertainty has largely been ignored (16). Yet recent methodological developments have been proposed to quantify and account for it (16, 39). It would be very interesting to investigate the impact of both types of state uncertainty in the data on bias and precision of eco-epidemiological parameters using simulations. Our framework and the codes that we provided could be used as a starting point.

### Conclusions and study prospects

In disease ecology, MECMR models improve precision and decrease bias of parameter estimates by incorporating unknown infection states instead of censoring them (18). Using simulations, we showed here that such models produce relatively unbiased and precise estimates of eco-epidemiological parameters even when the proportion of individuals in the U state is high, in conditions akin to the ones we have used here (see Table 1). As we provide a flexible framework, our study should be useful to disease ecologists, conservationists or wildlife managers who may wish to explore the potential bias in their parameter estimates *a posteriori*, by adjusting the values of input parameters, the number of occasions and the sample sizes (for an illustration, see the case study presented in the Supplementary Material Table S2). Our framework may be particularly useful to researchers constrained by non-invasive sampling approaches to assess the health status of animals [such as skin swabs in frogs e.g., (39)], as this may result in a high proportion of unknown infection states in data sets or heterogeneity in the assignment of infection states. Other types of disease dynamics can also be modeled, such as SI dynamics [e.g., (16, 23, 24). As uncertainty in disease ecology studies may in reality arise from several diverse processes, which extend well beyond state uncertainty, such as taxonomic crypticity or a mismatch of sampling and process scales [see (40) for a detailed review], researchers need to bear in mind that all potential sources of bias and imprecision, and their magnitude, should be considered prior to any study (40).

An interesting area for future research could be to explore bias and precision along a gradient of both heterogeneity in the detection and assignment of infection states, when state uncertainty increases. Here, we did not investigate the consequences of heterogeneity in detection probabilities among infection states. Such heterogeneity occurred for instance in a study of blue tits exposed to malaria, as infected individuals had higher detection probabilities than uninfected ones (23). In contrast, in house finches exposed to conjunctivitis (41) or in badgers to *Mycobacterium bovis* (16), non-infected individuals had higher detection/recapture probabilities than infected ones. It is possible that a high heterogeneity in the detection of S, I, or R states has a more important influence on bias than heterogeneity in the assignment of infection states. We provide here a flexible framework to explore such effects. Another promising methodological development to attenuate the effect of uncertainty on bias could be the use of a Bayesian approach to inform the prior on assignment probabilities.

## Data availability statement

The code to produce and analyze the simulated data sets for this study can be found on GitHub: https://github.com/oliviergimenez/sir_multievent/blob/master/biasandmse_on_R_with_recovery_probability.R (doi: 10.5281/zenodo.1286572).

## Author contributions

LM and SB developed the initial research idea. OG, SK-S, HH, and ME provided close support for improving it. J-DL provided specific guidance and expertise on CMR modeling. OG wrote the original R programming code and LM and SB edited it. SB wrote the paper and all other authors contributed critically to the drafts and gave final approval for publication.

### Conflict of interest statement

The authors declare that the research was conducted in the absence of any commercial or financial relationships that could be construed as a potential conflict of interest.

## Supplementary material

Supplementary Material Table S1 All values for bias and precision.

Supplementary Material Table S2 Case study of Serengeti spotted hyenas infected with canine distemper virus.
