# Supplementary material for: Robustness of Eco-Epidemiological Capture-Recapture Parameter Estimates to Variation in Infection State Uncertainty
Source: Front Vet Sci. 2018 Aug 28;5:197. doi: 10.3389/fvets.2018.00197 (PMC6121098; doi:10.3389/fvets.2018.00197)
Supplement: Supplementary Material Table S1 — All values for bias and precision. [file Presentation_1.pdf]

frontiers

Supplementary Material S1

Robustness of eco-epidemiological capture-recapture parameter estimates to variation in infection state uncertainty

Sarah Benhaiem[1,*,±], Lucile Marescot[1,2, ±], Heribert Hofer[1,3,4], Marion L. East[1], J-D Lebreton[2], Stephanie Kramer-Schadt[1,5],Olivier Gimenez[2]

[1] Department of Ecological Dynamics, Leibniz Institute for Zoo and Wildlife Research, Alfred-Kowalke-Strasse 17, D-10315 Berlin, Germany

[2] CEFE, CNRS, University Montpellier, University Paul Valéry Montpellier 3, EPHE, IRD, 1919 Route de Mende, 34293 Montpellier Cedex 5, France

[3] Department of Veterinary Medicine, Freie Universität Berlin, Oertzenweg 19b, 14195 Berlin, Germany

[4] Department of Biology, Chemistry, Pharmacy, Freie Universität Berlin, Takustrasse 3, 14195 Berlin, Germany

[5] Department of Ecology, Technische Universität Berlin, Rothenburgstr. 12, 12165 Berlin

*** Correspondence:**
benhaiem@izw-berlin.de

[±] contributed equally to this work

This document presents all bias and precision values obtained via the simulations presented in the main text (with **fictive** input parameter values).

Table S1: Overview of variation in bias (mean difference between the parameter value estimated via simulations and the input parameter value) and precision (minimum squared error, MSE) of parameter estimates in relation to an increasing infection state uncertainty (ranging between 20 and 90%) implemented as a decreasing assignment probability of infection states (ranging between 0.8 and 0.1). Bias and precision were calculated in data sets simulated under different scenarios of assigning infection states: homogeneous or heterogeneous assignment probabilities, in which the assignment of S (‘hetero S’), I (‘hetero I’) or R (‘hetero R’) states was reduced by 50% in comparison to the two other infection states. We used the following notations for the parameters: ϕ_S , ϕ_I and ϕ_R for the survival probability of individuals in susceptible, infected and recovered states, β for the infection probability, p_S, p_I and p_R for the detection probability and δ_S, δ_I and δ_R for the assignment probability of individuals in susceptible, infected and recovered states, respectively. Orange: cases where bias (in absolute value) was > 0.1 and ≤ 0.2 (light orange) and > 0.2 (dark orange). Blue: cases where precision was > 5 and ≤ 15 (light blue) and > 15 (dark blue).

Parameter	Increasing state uncertainty (decreasing assignment probability)																Scenario
	20% (0.8)		30% (0.7)		40% (0.6)		50% (0.5)		60% (0.4)		70% (0.3)		80% (0.2)		90% (0.1)		
	bias	MSE	bias	MSE	bias	MSE	bias	MSE	bias	MSE	bias	MSE	bias	MSE	bias	MSE	
ϕ_S	0.0	1.1	0.0	1.4	0.0	1.4	0.1	2.9	0.1	4.1	0.1	6.4	0.1	9.5	0.1	21.2	homogeneous
	0.0	1.6	0.0	1.8	0.0	2.4	0.1	3.7	0.1	5.9	0.1	7.0	0.1	11.2	0.2	44.1	hetero S
	0.0	1.1	0.0	1.6	0.0	1.9	0.1	2.6	0.1	4.1	0.1	4.5	0.1	6.6	0.1	18.1	hetero I
	0.0	1.1	0.0	1.7	0.0	1.5	0.1	3.4	0.1	3.2	0.1	4.7	0.1	6.4	0.2	23.5	hetero R
ϕ_I	0.0	0.1	0.0	0.2	0.0	0.3	0.0	0.3	0.0	0.6	0.0	0.6	0.0	1.0	0.0	0.5	homogeneous
	0.0	0.3	0.0	0.4	0.0	0.3	0.0	0.5	0.0	0.7	0.0	0.8	0.0	0.7	0.0	0.1	hetero S
	0.0	0.1	0.0	0.2	0.0	0.4	0.0	0.5	0.0	0.5	0.0	0.9	0.0	1.4	0.0	0.8	hetero I
	0.0	0.2	0.0	0.4	0.0	0.2	0.0	0.4	0.0	0.4	0.0	0.5	0.0	0.6	0.0	0.0	hetero R
ϕ_R	0.0	0.0	0.0	0.0	0.0	0.0	0.0	0.0	0.0	0.0	0.0	0.0	0.0	0.1	0.0	1.0	homogeneous
	0.0	0.0	0.0	0.0	0.0	0.0	0.0	0.0	0.0	0.0	0.0	0.0	0.0	0.2	0.0	0.7	hetero S
	0.0	0.0	0.0	0.0	0.0	0.0	0.0	0.0	0.0	0.0	0.0	0.0	0.0	0.1	0.0	0.5	hetero I
	0.0	0.0	0.0	0.0	0.0	0.0	0.0	0.0	0.0	0.0	0.0	0.0	0.0	0.4	0.1	2.5	hetero R
β	0.0	2.0	0.1	2.6	0.1	4.6	0.1	4.0	0.1	8.3	0.1	13.0	0.1	21.1	0.2	42.0	homogeneous
	0.1	3.5	0.1	4.1	0.1	5.8	0.1	8.9	0.1	14.4	0.1	21.3	0.2	35.0	0.3	66.8	hetero S
	0.0	1.5	0.1	2.5	0.1	5.2	0.1	6.9	0.1	8.9	0.1	10.9	0.1	17.0	0.2	34.5	hetero I
	0.1	2.8	0.1	3.5	0.1	4.3	0.1	6.1	0.1	8.8	0.1	13.1	0.1	22.4	0.2	43.1	hetero R
γ	0.0	0.9	0.0	1.0	0.0	1.9	0.0	2.4	-0.1	4.2	-0.1	7.8	-0.1	18.5	-0.2	27.3	homogeneous
	0.0	1.0	0.0	1.6	0.0	1.8	-0.1	4.2	-0.1	7.5	-0.1	9.3	-0.1	17.5	-0.1	12.0	hetero S
	0.0	0.7	0.0	1.5	0.0	2.2	-0.1	2.8	-0.1	4.2	-0.1	7.9	-0.1	19.6	-0.1	20.5	hetero I
	0.0	1.0	0.0	0.9	0.0	2.4	0.0	2.0	-0.1	6.9	-0.1	10.3	-0.1	13.3	-0.1	16.6	hetero R
p_S	0.0	0.5	0.0	0.1	0.0	1.4	0.1	2.8	0.1	13.2	0.2	22.8	0.2	38.1	0.2	42.0	homogeneous
	0.1	2.9	0.1	7.5	0.1	9.9	0.2	23.1	0.2	27.9	0.2	44.5	0.2	34.2	0.2	39.3	hetero S
	0.0	1.8	0.0	0.1	0.0	1.9	0.1	3.1	0.1	9.9	0.2	27.3	0.2	44.3	0.2	48.8	hetero I
	0.0	1.3	0.0	0.1	0.0	0.7	0.0	2.1	0.1	13.5	0.2	26.1	0.2	37.2	0.2	39.4	hetero R
p_I	-0.1	3.6	-0.1	4.8	-0.1	6.9	-0.1	7.7	-0.1	14.0	-0.1	20.2	-0.2	28.4	-0.2	33.8	homogeneous
	-0.1	5.6	-0.1	6.7	-0.1	8.3	-0.1	13.2	-0.1	17.9	-0.1	20.4	-0.1	22.1	-0.2	32.0	hetero S
	-0.1	3.1	-0.1	5.3	-0.1	7.6	-0.1	9.5	-0.1	14.7	-0.1	16.8	-0.2	27.0	-0.2	32.2	hetero I
	-0.1	4.7	-0.1	5.7	-0.1	7.0	-0.1	10.8	-0.1	14.2	-0.1	18.4	-0.2	27.6	-0.2	39.4	hetero R
p_R	0.0	0.0	0.0	0.0	0.0	0.0	0.0	0.0	0.0	0.0	0.0	0.0	0.0	0.1	0.0	0.2	homogeneous
	0.0	0.0	0.0	0.0	0.0	0.0	0.0	0.0	0.0	0.0	0.0	0.0	0.0	0.1	0.0	0.5	hetero S
	0.0	0.0	0.0	0.0	0.0	0.0	0.0	0.0	0.0	0.0	0.0	0.0	0.0	0.0	0.0	0.3	hetero I
	0.0	0.0	0.0	0.0	0.0	0.0	0.0	0.0	0.0	0.0	0.0	0.0	0.0	0.1	0.0	0.3	hetero R
δ_S	0.0	0.2	0.0	0.8	0.0	1.4	0.0	2.0	-0.1	4.9	-0.1	6.9	-0.1	12.1	-0.1	20.7	homogeneous
	0.0	0.4	0.0	0.5	0.0	0.5	0.0	1.3	-0.1	3.8	-0.1	8.2	-0.1	10.3	-0.2	24.3	hetero S
	0.0	0.0	0.0	0.3	0.0	1.1	0.0	0.8	-0.1	4.6	-0.1	8.7	-0.1	11.4	-0.2	24.2	hetero I
	0.0	0.2	0.0	0.5	0.0	1.6	-0.1	4.2	-0.1	5.7	-0.1	9.0	-0.1	16.9	-0.1	19.9	hetero R
δ_I	0.0	0.0	0.0	0.1	0.0	0.1	0.0	0.3	0.0	0.5	0.0	1.0	-0.1	2.9	-0.1	6.4	homogeneous
	0.0	0.1	0.0	0.4	0.0	0.5	0.0	1.2	0.0	1.7	-0.1	3.0	-0.1	6.7	-0.1	8.9	hetero S
	0.0	0.1	0.0	0.1	0.0	0.2	0.0	0.4	0.0	0.6	0.0	0.8	-0.1	2.6	-0.1	5.6	hetero I
	0.0	0.1	0.0	0.1	0.0	0.3	0.0	0.3	0.0	0.8	0.0	2.0	-0.1	3.4	-0.1	8.0	hetero R
δ_R	0.0	0.0	0.0	0.0	0.0	0.0	0.0	0.0	0.0	0.0	0.0	0.0	0.0	0.0	0.0	0.3	homogeneous
	0.0	0.0	0.0	0.0	0.0	0.0	0.0	0.0	0.0	0.0	0.0	0.0	0.0	0.0	0.0	0.8	hetero S
	0.0	0.0	0.0	0.0	0.0	0.0	0.0	0.0	0.0	0.0	0.0	0.0	0.0	0.0	0.0	0.4	hetero I
	0.0	0.0	0.0	0.0	0.0	0.0	0.0	0.0	0.0	0.0	0.0	0.1	0.0	0.3	-0.1	9.7	hetero R

bias

- $\leq |0.1|$
- $> |0.1|$ and $\leq |0.2|$
- $> |0.2|$

precision

- ≤ 5
- >5 and ≤ 15
- > 15
