# Supplementary material for: Robustness of Eco-Epidemiological Capture-Recapture Parameter Estimates to Variation in Infection State Uncertainty
Source: Front Vet Sci. 2018 Aug 28;5:197. doi: 10.3389/fvets.2018.00197 (PMC6121098; doi:10.3389/fvets.2018.00197)
Supplement: Supplementary Material Table S2 — Case study of Serengeti spotted hyenas infected with canine distemper virus. [file Presentation_2.pdf]

Supplementary Material S2

Robustness of eco-epidemiological capture-recapture parameter estimates to variation in infection state uncertainty

Sarah Benhaiem[1,*,±], Lucile Marescot[1,2, ±], Heribert Hofer[1,3,4], Marion L. East[1], J-D Lebreton[2], Stephanie Kramer-Schadt[1,5],Olivier Gimenez[2]

[1] Department of Ecological Dynamics, Leibniz Institute for Zoo and Wildlife Research, Alfred-Kowalke-Strasse 17, 10315 Berlin, Germany

[2] CEFE, CNRS, University Montpellier, University Paul Valéry Montpellier 3, EPHE, IRD, 1919 Route de Mende, 34293 Montpellier Cedex 5, France

[3] Department of Veterinary Medicine, Freie Universität Berlin, Oertzenweg 19b, 14195 Berlin, Germany

[4] Department of Biology, Chemistry, Pharmacy, Freie Universität Berlin, Takustrasse 3, 14195 Berlin

[5] Department of Ecology, Technische Universität Berlin, Rothenburgstr. 12, 12165 Berlin

*** Correspondence:**
benhaiem@izw-berlin.de

[±] contributed equally to this work

This document presents the results obtained for the **case study** of **spotted hyenas** (*Crocuta crocuta*) infected with **canine distemper virus** in the Serengeti National Park in Tanzania.

1 Supplementary Data

To estimate empirically survival and infection probabilities we developed a simple SIR-MECMR model (Pradel 2005) fitted in E-SURGE 1.9.0. (Choquet & Nogue 2011) to a two decades (1990-2010) data set of free-ranging female spotted hyenas infected with a highly virulent strain of canine distemper virus (CDV), adapted to non-canid species (Nikolin et al. 2017). This CDV strain caused a substantial decline in the African lion (*Panthera leo*) population in Serengeti National Park (Roelke-Parker et al. 1996) during an epidemic in 1993/1994, and reduced the growth rate of the spotted hyena population in the Park, by decreasing the survival of juveniles (Marescot et al. 2018, Benhaiem et al. under review).

The data set comprises 625 females in three large clans and is fully described elsewhere (Marescot et al. 2018, Benhaiem et al. under review). Briefly, we synthesized multiple systematic observations of individuals at communal and birth dens within their clan territories into single yearly summaries. When an individual was detected a given year, it was assigned an infection state, accounting for uncertainty in the infection state. The three infection states were ‘susceptible’ (S), ‘infected’ (I) and ‘recovered’ (R).

We used the results of three diagnostic procedures to assign these states; 1) RT-PCR screening for the presence or absence of CDV RNA in samples, 2) CDV antibody titres in serum and 3) the observation of clinical signs associated with CDV infection in hyenas, and the secondary infections it causes in this species (Haas et al. 1996). How infection states were assigned based on the results of these procedures is fully detailed in Marescot et al. 2018. CDV infection could occur only once in life because hyenas develop life-long immunity to CDV if they survive the infection. The original model presented in Marescot et al. 2018 and Benhaiem et al. under review included these three infection states, but also four demographic states and two social states. To simplify the simulation study and assess bias and precision of parameter estimates in relation to state uncertainty, we considered a simple SIR model, structured with parameter estimates constant over time and across demographic and social states, but with the survival probability potentially varying between S, I and R states.

The estimated parameters (Table S1) were then used as input to simulate data sets and quantify parameter bias and precision exactly as described in the Methods section of the main text. Please note that because CDV induces lifelong immunity, the recovery probability (γ) was set to 1 here.
Supplementary Figures and Tables

Table S1. Maximum likelihood estimates (M.L.E) (± S.E.) of annual probabilities of surviving (ϕ), becoming infected (β) and detection (p) obtained via E-SURGE 1.9.0 and further used as input parameter values to simulate datasets.

**Parameter**	**Description**	**M.L.E ± S.E.**
ϕ_S	Survival probability of susceptible	0.96 ± 0.02
ϕ_I	Survival probability of infected	0.53 ± 0.05
ϕ_R	Survival probability of recovered	0.84 ± 0.01
β	Infection probability	0.40 ± 0.04
p	Detection probability	0.99 ± 0.00

Table S2. Overview of variation in bias and precision of parameter estimates in relation to increasing infection state uncertainty (ranging between 20 and 90%), i.e. a decreasing assignment probability of infection states (ranging between 0.8 and 0.1). Bias was the mean difference between the value obtained from simulations and the input parameter value by the MECMR model fitted in E-SURGE, precision was the minimum squared error (MSE). Bias and precision were calculated for data sets simulated under different scenarios: homogeneous or heterogeneous assignment probabilities (in which the assignment of S (hetero S), I (hetero I) or R (hetero R) states was reduced by 50% in comparison to the two other infection states. We used the following notations for the parameters: ϕ_S, ϕ_I and ϕ_R for the survival probability of individuals in susceptible, infected and recovered states, β for the infection probability, p_S, p_I and p_R for the detection probability and δ_S, δ_I and δ_R for the assignment probability of individuals in susceptible, infected and recovered states, respectively. Orange color highlights cases where bias (in absolute value) was > 0.05 and ≤ 0.10 (light orange) and where it was > 0.10 (dark orange). Blue color highlights cases where precision was > 1 and ≤ 10 (light blue) and cases where it was > 10 (dark blue).

Parameter	Increasing state uncertainty (Decreasing assignment probability)																Scenario
	20% (0.8)		30% (0.7)		40% (0.6)		50% (0.5)		60% (0.4)		70% (0.3)		80% (0.2)		90% (0.1)		
	bias	MSE	bias	MSE	bias	MSE	bias	MSE	bias	MSE	bias	MSE	bias	MSE	bias	MSE	
ϕ_S	-0.01	0.06	0.00	0.00	0.00	0.01	-0.01	0.10	-0.02	0.38	-0.02	0.23	-0.01	0.17	-0.01	0.06	homogeneous
	-0.01	0.04	0.00	0.01	0.00	0.02	-0.01	0.09	-0.02	0.33	-0.01	0.22	-0.01	0.16	-0.01	0.03	hetero S
	-0.01	0.05	0.00	0.01	0.00	0.02	-0.01	0.11	-0.02	0.31	-0.02	0.28	-0.01	0.18	-0.01	0.04	hetero I
	0.00	0.02	-0.01	0.07	-0.01	0.14	-0.01	0.15	-0.01	0.14	-0.01	0.16	-0.01	0.04	0.00	0.01	hetero R
ϕ_I	0.00	0.02	0.00	0.00	0.00	0.00	0.00	0.02	0.01	0.06	0.01	0.03	0.01	0.04	0.00	0.01	homogeneous
	0.00	0.01	0.00	0.00	0.00	0.00	0.00	0.01	0.01	0.05	0.01	0.03	0.01	0.03	0.00	0.02	hetero S
	0.00	0.01	0.00	0.00	0.00	0.00	0.00	0.01	0.01	0.05	0.01	0.05	0.01	0.03	0.00	0.02	hetero I
	0.00	0.01	0.00	0.02	0.01	0.04	0.00	0.02	0.01	0.03	0.01	0.03	0.00	0.01	0.00	0.02	hetero R
ϕ_R	0.00	0.00	0.00	0.00	0.00	0.00	0.00	0.00	0.00	0.00	0.00	0.00	0.00	0.00	0.00	0.00	homogeneous
	0.00	0.00	0.00	0.00	0.00	0.00	0.00	0.00	0.00	0.00	0.00	0.00	0.00	0.00	0.00	0.00	hetero S
	0.00	0.00	0.00	0.00	0.00	0.00	0.00	0.00	0.00	0.00	0.00	0.00	0.00	0.00	0.00	0.00	hetero I
	0.00	0.00	0.00	0.00	0.00	0.00	0.00	0.00	0.00	0.00	0.00	0.00	0.00	0.00	0.00	0.00	hetero R
β	-0.01	0.03	0.00	0.00	0.00	0.01	-0.01	0.06	-0.01	0.14	-0.01	0.12	-0.01	0.11	-0.01	0.09	homogeneous
	0.00	0.02	0.00	0.01	0.00	0.02	-0.01	0.05	-0.01	0.18	-0.01	0.10	-0.01	0.10	-0.01	0.04	hetero S
	-0.01	0.03	0.00	0.01	0.00	0.01	-0.01	0.05	-0.01	0.14	-0.01	0.15	-0.01	0.13	0.00	0.02	hetero I
	0.00	0.02	-0.01	0.03	-0.01	0.08	-0.01	0.06	-0.01	0.07	-0.01	0.10	-0.01	0.05	0.00	0.00	hetero R
p_S	0.00	0.00	0.00	0.00	0.00	0.00	0.00	0.00	0.00	0.00	0.00	0.00	0.00	0.00	0.00	0.00	homogeneous
	0.00	0.00	0.00	0.00	0.00	0.00	0.00	0.00	0.00	0.00	0.00	0.00	0.00	0.01	0.00	0.00	hetero S
	0.00	0.00	0.00	0.00	0.00	0.00	0.00	0.00	0.00	0.00	0.00	0.00	0.00	0.00	0.00	0.01	hetero I
	0.00	0.00	0.00	0.00	0.00	0.00	0.00	0.00	0.00	0.00	0.00	0.00	0.00	0.00	0.00	0.01	hetero R
p_I	0.02	0.27	0.01	0.05	0.01	0.05	0.02	0.38	0.04	1.32	0.03	0.83	0.02	0.60	0.02	0.35	homogeneous
	0.02	0.23	0.01	0.04	0.01	0.08	0.02	0.41	0.03	1.17	0.03	0.89	0.02	0.58	0.02	0.26	hetero S
	0.02	0.25	0.01	0.03	0.01	0.07	0.02	0.43	0.03	1.10	0.03	1.00	0.03	0.64	0.02	0.34	hetero I
	0.01	0.12	0.02	0.31	0.02	0.58	0.03	0.64	0.03	0.63	0.02	0.56	0.02	0.27	0.02	0.39	hetero R
p_R	0.00	0.00	0.00	0.00	0.00	0.00	0.00	0.00	0.00	0.00	0.00	0.00	0.00	0.00	0.00	0.00	homogeneous
	0.00	0.00	0.00	0.00	0.00	0.00	0.00	0.00	0.00	0.00	0.00	0.00	0.00	0.00	0.00	0.00	hetero S
	0.00	0.00	0.00	0.00	0.00	0.00	0.00	0.00	0.00	0.00	0.00	0.00	0.00	0.00	0.00	0.00	hetero I
	0.00	0.00	0.00	0.00	0.00	0.00	0.00	0.00	0.00	0.00	0.00	0.00	0.00	0.00	0.00	0.00	hetero R
δ_S	0.00	0.00	0.00	0.00	0.00	0.00	0.00	0.00	0.00	0.01	0.00	0.00	0.00	0.01	0.00	0.01	homogeneous
	0.00	0.00	0.00	0.00	0.00	0.00	0.00	0.00	0.00	0.01	0.00	0.01	0.00	0.01	0.00	0.00	hetero S
	0.00	0.00	0.00	0.00	0.00	0.00	0.00	0.00	0.00	0.00	0.00	0.01	0.00	0.00	0.00	0.00	hetero I
	0.00	0.00	0.00	0.00	0.00	0.00	0.00	0.00	0.00	0.00	0.00	0.00	0.00	0.00	0.00	0.00	hetero R
δ_I	0.00	0.00	0.00	0.00	0.00	0.00	0.00	0.00	0.00	0.00	0.00	0.00	0.00	0.00	0.00	0.00	homogeneous
	0.00	0.00	0.00	0.00	0.00	0.00	0.00	0.00	0.00	0.00	0.00	0.00	0.00	0.00	0.00	0.00	hetero S
	0.00	0.00	0.00	0.00	0.00	0.00	0.00	0.00	0.00	0.00	0.00	0.00	0.00	0.00	-0.01	0.04	hetero I
	0.00	0.00	0.00	0.00	0.00	0.00	0.00	0.00	0.00	0.00	0.00	0.00	0.00	0.00	0.00	0.00	hetero R
δ_R	0.00	0.00	0.00	0.00	0.00	0.00	0.00	0.00	0.00	0.00	0.00	0.00	0.00	0.01	0.00	0.00	homogeneous
	0.00	0.00	0.00	0.00	0.00	0.00	0.00	0.00	0.00	0.00	0.00	0.00	0.00	0.00	0.00	0.00	hetero S
	0.00	0.00	0.00	0.00	0.00	0.00	0.00	0.00	0.00	0.00	0.00	0.00	0.00	0.00	0.00	0.00	hetero I
	0.00	0.00	0.00	0.00	0.00	0.00	0.00	0.00	0.00	0.00	0.00	0.00	0.00	0.00	0.00	0.00	hetero R

bias

- ≤ |0.05|
- > |0.05| and ≤ |0.10|
- > |0.10|

precision

- ≤ 1
- >1 and ≤ 10
- > 10

2 Supplementary results

Our results show that CDV infection caused an important decrease in the survival of spotted hyenas (Table S1). We found with the model accounting for parameters varying between S, I and R states that the survival probability was reduced by 43% between susceptible and infected individuals and increased by 31% between infected and recovered ones. We found that individuals would become infected in a given year with a probability of 0.40 ± 0.04 (Table S1).

In this case study, we observed a slight increase in bias and a slight decrease in precision as state uncertainty increased from 20 to 90 %, for all parameters and regardless of the scenario considered (see Table S2). The detection probability of infected individuals was the parameter estimate that departed the most from the true parameter value. Its maximum bias was reached at 60 % of state uncertainty with a value of 0.036. The most underestimated parameter was the survival probability of susceptible individuals, which reached a maximum negative bias of -0.0195 at 60% of state uncertainty in the homogeneous scenario. In terms of precision, the most sensitive parameter was the detection of infected individuals p_I. The highest MSE value of p_I was 1.3 and was found at a level of state uncertainty equal to 60%, generated with homogeneous assignment probabilities.

3 References

Benhaiem, S., Marescot, L., East, M.L., Kramer-Schadt, S., Gimenez, O., Lebreton, J.D. and Hofer, H. Slow recovery from a disease epidemic in a keystone social carnivore. Under review.

Choquet, R. and Nogue, E. (2011) E-SURGE 1.8 user's manual. CEFE, UMR 5175, Montpellier, France.

Haas, L., Hofer, H., East, M., Wohlsein, P., Liess, B. and Barrett, T. (1996). Canine distemper virus infection in Serengeti spotted hyaenas. Vet. Microbiol. 49, 147-152. doi: 10.1016/0378-1135(95)00180-8

Marescot, L., Benhaiem, S, Gimenez, O., Hofer, H., Lebreton, J-D, Olarte-Castillo, X.A., Kramer-Schadt, S., East, M.L. (2018). Social status mediates the fitness costs of infection with canine distemper virus in Serengeti spotted hyenas. Funct. Ecol. 32, 1237-1250. doi: 10.1111/1365-2435.13059

Nikolin, V.N., Olarte-Castillo, X.A., Osterrieder, N., Hofer, H., Dubovi, E., Mazzoni et al. (2017) Canine distemper virus in the Serengeti ecosystem: molecular adaptation to different carnivore species. Mol. Ecol. 26, 2111-2130. doi: 10.1111/mec.13902

Pradel, R. (2005) Multievent: an extension of multistate capture–recapture models to uncertain states. Biometrics 61, 442-447. doi: 10.1111/j.1541-0420.2005.00318.x

Roelke-Parker, M. E., Munson, L., Packer, C., Kock, R., Cleaveland, S., Carpenter, M. et al. (1996). A canine distemper virus epidemic in Serengeti lions (*Panthera leo*). Nature, 379: 441-445. doi: 10.1038/379441a0
